# Characterizing the Documentation of Time-Limited Trials in Goals of Care Notes

**DOI:** 10.1016/j.jpainsymman.2025.04.007

**Published:** 2025-04-17

**Authors:** Gina M. Piscitello, Robert M. Arnold, Jane O. Schell, Jacqueline M. Kruser

**Affiliations:** Division of General Internal Medicine, Section of Palliative Care and Medical Ethics, University of Pittsburgh, Pittsburgh, Pennsylvania, USA; Palliative Research Center, University of Pittsburgh, Pittsburgh, Pennsylvania, USA; Brookdale Department of Geriatrics and Palliative Medicine, Icahn School of Medicine at Mount Sinai, New York City, New York, USA; Division of General Internal Medicine, Section of Palliative Care and Medical Ethics, University of Pittsburgh, Pittsburgh, Pennsylvania, USA; Palliative Research Center, University of Pittsburgh, Pittsburgh, Pennsylvania, USA; Department of Medicine, Division of Allergy, Pulmonary, and Critical Care, University of Wisconsin School of Medicine and Public Health, Madison, Wisconsin, USA

**Keywords:** Time-limited trial, goals of care, clinician-patient communication

## Abstract

**Context.:**

Time-limited trials (TLTs) are a collaborative plan among clinicians, patients, and surrogates to use life-sustaining therapy for a defined duration, after which the response to therapy informs the decision to either continue care focused on recovery or transition to comfort-focused care.

**Objectives.:**

To evaluate 1) how often goals of care (GOC) notes document TLT use; 2) what patient and clinician characteristics are associated with documented TLTs; and 3) how TLTs are described in GOC documentation.

**Methods.:**

We conducted a retrospective cross-sectional study of documented standardized GOC template notes for seriously ill hospitalized adult patients across 21-hospitals between 2021 and 2023. We evaluated notes using descriptive statistics paired with qualitative, directed content analysis.

**Results.:**

Of 5475 GOC-template notes, we found reference to a TLT in 1% (*n* = 69/5475). Patients with TLT documentation were younger (72 vs 76 years, *P* = 0.0221), more likely to be self-pay or uninsured (7% vs. 2%, *P* = 0.0309), more likely to die in the hospital (54% vs. 27%, *P* < 0.0001) or discharge to a long-term acute care hospital (15% vs. 1%, *P* < 0.0001), when compared to patients with no such documentation. Notes with TLT documentation were more likely to document patient prognosis (94% vs. 79%, *P* = 0.0009) and less likely to document an accompanying change in patient code status (22% vs. 36%, *P* = 0.0117).

**Conclusions.:**

TLTs are rarely documented within a standardized GOC note for seriously ill patients. Future research is needed to identify and address reasons for low documentation of TLTs in the EHR.

## Introduction

A time-limited trial (TLT) is a collaborative plan made by patients, surrogates, and clinicians to use life-sustaining therapies for a specific duration, followed by an assessment of the patient’s response to determine whether to continue, change, or stop these therapies.^1^ Many clinicians use TLTs in practice, and professional societies endorse this approach to navigate prognostic uncertainty for patients with serious illness.^1–8^

After a TLT plan is made, its documentation in the electronic health record (EHR) is important so that it is disseminated within the large, rotating, interprofessional teams who care for patients with acute, serious illness. In addition, transparent and clear TLT documentation is necessary to maintain trust about this collaborative plan, as patients and surrogates have access to view EHR notes.^9^ Indeed, a consensus report from the American Thoracic Society recently established EHR documentation as an essential element for conducting a TLT. Despite this importance, little is known about the extent to which and how well TLTs are currently documented in EHRs. The only evidence, to our knowledge, is a single-center observational study which found that 64% of TLTs identified as such by direct query to the intensive care unit (ICU) physician were documented at least once in the EHR.^10^

To improve our understanding of TLT documentation in the EHR, this study aims to evaluate 1) how often goals of care (GOC) notes document the use of a TLT; 2) what patient and clinician characteristics are associated with documented TLTs; and 3) how TLTs are described in this documentation. Our overarching goal is to use this understanding to improve the documentation and dissemination of TLT plans.

## Methods

### Overview and Study Design

We conducted a retrospective cross-sectional study to evaluate a standardized goals of care (GOC) template note, which was implemented in July 2021 across 21 hospitals within one large medical system in the northeast United States. These hospitals include academic quaternary care centers, and urban, suburban and rural community hospitals. Approval for this study was obtained by the University of Pittsburgh Medical Center Quality Improvement Review Committee, project ID 2629. We followed Strengthening the Reporting of Observational Studies in Epidemiology (STROBE) guidelines to present our results.^11^

### Study Population and Setting

We identified all seriously ill patients admitted to 21 hospitals between July 2021 and March 2023, using an artificial intelligence mortality model created by the health system that predicted patients with greater than 30% risk of 90-day mortality, which has previously been described.^12,13^ Given that TLTs are framed as an approach to address prognostic uncertainty, this hospitalized patient population with at least moderate risk of near-term death represents those patients for whom TLTs are potentially used. We included patients admitted to ICUs and general care floors, as life-sustaining therapies and TLTs can be discussed in both locations (e.g., artificial nutrition and hydration or preemptive decisions preceding an ICU stay).

In this identified patient population, we then abstracted the first GOC note documented for patients in this cohort, using a standardized, system-wide GOC template note. All hospitalized patient admissions were eligible for inclusion in this study. For patients with repeat admissions during the study period, each unique admission was included in the study analysis. Health system clinicians were encouraged to use the GOC template note to document all GOC conversations including discussion about 1) surrogate identification, 2) prognosis discussion, 3) patient values, 4) decisions made, and/or 5) discharge plans and next steps.^13^ The standardized GOC template note was used frequently in this population during the study period, with over 70% GOC template note documentation for patients with >60% risk of 90-day mortality and 28% for patients with 30–60% risk of 90-day mortality.^13^

### Demographics

From the EHR, we collected patient demographics including age, sex, race, primary insurance, and area deprivation index.^14^ Patient race was self-identified and categorized into three categories: Black, White and Other based on EHR determination. Patients with “Other” race included all patients that did not self-identify as Black or White. Insurance was categorized into five categories: Medicaid, Medicare, no insurance, private, and VA insurance. We collected clinical data including Charlson Comorbidity Index, dementia diagnosis, ICU admission, and specialty palliative consult. We collected patient outcome measures including length of hospital stay, 30-day readmission, in-hospital mortality and discharge location.

### Time-Limited Trials Within the GOC Template Note

The GOC template is comprised of check boxes and free response entries, as has been described.^13^ It includes prompts to document attendance at the GOC meeting, surrogate information, prognosis, patient values, and decisions made. One prompt asks the clinician to respond to the question, “Change in care plan included” followed by categorical options. One such option is “Time-limited trial.” Documentation of a TLT in our study was defined by selection of this “Time-limited trial” option in the GOC template note. For clinician who selected the “Time-limited trial” option, additional prompts include the “Date to check in on success of the trial” and a free response box “Goals discussed that would show the trial successful” (eFig. 1).

### Statistical Analysis

We described continuous variables as median and interquartile range (IQR) and categorical variables as quantity and percent. We compared continuous variables using t-test and categorical variables using Fisher’s Exact or Chi-squared tests as appropriate. We used qualitative, directed content analysis to evaluate the free response boxes in the GOC template note and assessed how this data aligned with the consensus report on the essential elements for conducting a TLT.^1^

## Results

We identified 5475 GOC template notes documented for 47,699 seriously ill adult patient admissions across twenty-one hospitals. Patients with GOC template notes had median age 76, were 51% male, 13% nonwhite, and 81% with Medicare insurance. We found documentation referring to a TLT in 1% (*n* = 69/5475) of all GOC template notes. Sixty-eight percent of GOC notes documenting a TLT were for patients admitted to an ICU (*n* = 47). For the subset of GOC notes for patients admitted to an ICU, TLT documentation was identified in 2% (*n* = 47/2098).

### Comparison of Patients With and Without TLT Documentation

Patients with TLT documentation were younger (72 vs 76 years, *P* = 0.0221) and more likely to be self-pay or uninsured (7% vs. 2%, *P* = 0.0309) when compared to patients with GOC notes lacking such documentation. Patients with TLT documentation were also more likely to be admitted to the intensive care unit (67% vs. 37%, *P* < 0.0001), more likely to die in the hospital (54% vs. 27%, *P* < 0.0001) and more likely to discharge to a long-term acute care hospital (15% vs. 1%, *P* < 0.0001). Patients with TLT documentation had longer median hospital length of stays (10 vs. 8 days, *P* = 0.0368) and lower 30-day readmission rates (6% vs. 15%, *P* = 0.0382) (Table 1).

Documented GOC discussions that resulted in TLTs were more likely to be held by primary team clinicians only without specialty palliative clinicians present (51% vs. 39%, *P* = 0.0470), less likely to include specialty palliative medicine clinicians alone without the presence of primary team clinicians (30% vs. 43%, *P* = 0.0370), and less likely to include the patient (42% vs. 59%, *P* = 0.0087). Notes with TLT documentation were more likely to also include patient prognosis (94% vs. 79%, *P* = 0.0009) and less likely to document an accompanying change in patient code status (22% vs. 36%, *P* = 0.0117) (Table 2).

### Incorporation of Essential TLT Elements Into GOC Documentation

GOC notes documenting TLTs regularly recorded essential TLT elements including patient goals and priorities (97%), patient prognosis (79%), TLT duration (96%), clinical criteria for improvement or deterioration (99%), acceptable therapies during the TLT (90%), and potential decisions at the end of the TLT (70%). Specific prognostic estimates were provided in 38% of GOC notes and varied from hours to days (16%), days to weeks (13%) and weeks to months (9%). Specific documented clinical criteria for improvement or deterioration most frequently included improvement in mentation (23%) or respiratory status (17%), or decreased life-support requirements (13%). Essential TLT elements less regularly recorded in GOC notes included documentation of patient or surrogate agreement to the TLT (46%) and documentation of a planned future meeting between clinicians, patients and surrogates to discuss patient’s response to therapy (51%) (Table 3).

## Discussion

In this cross-sectional study of standardized GOC notes for seriously ill, hospitalized patients across 21 hospitals, we found low frequency of TLT documentation. Given that TLTs are primarily used to navigate the use of life-sustaining treatment, it is not surprising that TLT documentation was more frequent in patients with higher severity of illness (i.e., who were admitted to an ICU, who ultimately died in the hospital, or who were discharged to a long-term acute care hospital). Yet, we also found that TLTs are not exclusively an ICU approach—with 32% of all documented TLTs occurring in patients on general care floors. TLTs were more frequently documented in populations who were uninsured, which may signify that GOC decisions about LST may differ for this population, as has been identified in past research.^15^ When TLTs were documented, we found that many of the essential, consensus-based elements were included, such as the duration of the trial and the clinical criteria of improvement or deterioration. Yet, fewer than half of the notes addressed patient or surrogate agreement to the TLT or scheduled a future meeting for reassessment.

Our finding that only 1% of all GOC notes and only 2% of GOC notes written for ICU patients had documentation of a TLT was surprising. Previous research identified TLT rates in similar samples of seriously ill patients to be 8–27%.^5,6,10^ These estimates of TLT frequency, however, were based on direct observation of care delivery (e. g., audio recorded family meetings, direct clinician query) and not EHR data.^5,6,10^ Thus, one potential explanation for our finding is that TLTs were used more often in this study sample but not always documented. The only prior work examining TLT documentation found that 64% of TLTs identified directly by ICU physicians had accompanying documentation in the EHR.^10^ This potential lack of adequate documentation of TLTs is concerning, given the importance of EHR documentation to disseminate this care plan across the large, interprofessional, rotating teams of clinicians as well as patients and surrogates who can read these notes.

One factor that may contribute to lower documentation of TLTs is lack of clinician clarity about whether the patient’s care plan constituted a TLT. While a consensus definition for what constitutes a TLT now exists, this definition was published following the end date of our study period.^1^ A second factor that may contribute to lower documentation of TLTs is the higher documentation burden associated with these plans of care. For example, within the GOC template note assessed in this study, clinicians were prompted to document essential TLT elements, such as date to reassess the TLT trial. While these prompts may have the benefit of encouraging clinicians to thoroughly document TLT plans, it may consequentially deter clinicians who are already burdened by EHR documentation requirements from accurately describing a TLT.^16^ In a recent single-center study where TLTs were identified by direct query to the ICU physician, the majority of TLTs (64%) were referenced at least once in the EHR.^10^ These findings highlight the need for interventions to support documentation of all TLTs that are taking place.

A third potential explanation for the low frequency of TLT documentation in our study is the low frequency of TLT use in this health system. However, we suspect this is less likely given the systems’ educational efforts to encourage clinicians to consider using TLTs and documenting TLTs using the GOC template note. If this explanation is true, however, this suggests substantial variation in the adoption of TLTs across the United States (ranging from 1% in this study up to 27% in other studies). This finding would fit with institutional-level variation in the use of TLTs previously described in an ethnographic study of two different ICUs, but our findings extend the extent of this variation.^5^

While many essential elements for TLTs were included in GOC notes, less than half of notes documented that those patients or surrogates were provided an opportunity to agree to or decline to participate in a TLT. It is possible clinicians did ensure that patients or surrogates agreed to this plan but were less likely to document about this step because 1) a specific prompt encouraging documentation of agreement did not exist in the GOC template or 2) because clinicians assumed this agreement was implied if they documented that a TLT decision was made. In contrast, it is plausible that clinicians did not fully engage patients or their surrogates in the TLTs process due to clinician belief that pursuing a TLT is a medical decision that does not require consent of a patient or surrogate. Reasons for low documentation of patient or surrogate engagement within the TLT planning process should be explored in future studies.

### Strengths and Limitations

While a strength of this study is our multicenter examination of 21 hospitals within one health system, our findings may not be generalizable to hospitals that use a different GOC template note or do not use a template to document GOC notes. This study only assessed for documentation of TLT decisions within the first GOC note documented for a seriously ill patient and did not assess other locations where TLT decisions may be documented, such as daily progress notes. This study only assessed documentation of TLTs and did not compare this documentation with what took place during GOC conversations. It is important that future research assesses how documentation of TLTs within GOC notes aligns with how TLTs are described during GOC conversations.

## Conclusions

This multicenter, cross-sectional study of EHR documentation found that TLTs are rarely documented within a standardized GOC note for seriously ill patients, despite an explicit prompt within the note. This finding may represent a substantial gap between what is being done in clinical practice and what is being documented in the EHR or very low TLT adoption in this health system compared to others. Given the importance of TLT documentation for its success as a longitudinal plan of care, future work should focus on improving this documentation.

## Supplementary Material

1

## Figures and Tables

**Table 1:** Patent Characteristics by Documentation of Time-Limited Trial Within Goals of Care Notes

Patient Characteristics	All Notes(*N* = 5475)	Notes With Documentation of a Time-Limited Trial (*n* = 69)	Notes Without Documentation of a Time-Limited Trial (*n* = 5406)
Age (median, IQR)	76 (66–85)	72 (64–84)	76 (66–85)
Sex			
Female	2655 (48)	30 (43)	2625 (49)
Male	2820 (51)	39 (57)	2781 (51)
Race			
Black	598 (11)	8 (12)	590 (11)
Other	136 (2)	2 (3)	134 (2)
White	4741 (87)	59 (85)	4682 (87)
Primary insurance			
Medicaid	342 (6)	6 (9)	336 (6)
Medicare	4424 (81)	50 (72)	4374 (81)
No insurance	140 (3)	5 (7)	135 (2)
Private	478 (9)	7 (10)	471 (9)
VA insurance	91 (2)	1 (1)	90 (2)
Area deprivation index			
80 and higher	1123 (21)	16 (23)	1107 (20)
Charlson comorbidity index (median, IQR)	3 (1–4)	2 (1–4)	3 (1–4)
Dementia diagnosis	735 (13)	9 (13)	764 (14)
Intensive care unit admission	2145 (39)	46 (67)	2099 (39)
Length of hospital stay (median, IQR)	8 (4–14)	10 (4–18)	8 (4–14)
30-day readmission	778 (14)	4 (6)	802 (15)
Discharge			
Died in hospital	1503 (27)	37 (54)	1466 (27)
Hospice	1179 (22)	10 (15)	1169 (22)
Home	1472 (27)	8 (12)	1464 (27)
Long-term acute care hospital	53 (1)	10 (15)	50 (1)
Skilled nursing facility	1023 (19)	3 (4)	1013 (19)
Inpatient rehab	160 (3)	1 (1)	159 (3)
Specialty palliative consult	4227 (77)	47 (68)	4180 (77)
Hospital location			
Urban academic	2827 (52)	29 (42)	2798 (52)
Nonrural community	2516 (46)	36 (52)	2480 (46)
Rural community	132 (2)	4 (6)	128 (2)

Quantity (percent) unless otherwise indicated.

Area deprivation index ranges from 0 to 100 with 100 indicating areas of highest disadvantage.

IQR = interquartile range.

**Table 2:** Comparison of Goals of Care Note Content by Time-Limited Trial Documentation Status

Content of Goals of Care Notes	All (*N* = 5475)	Notes With Documentation of a Time-Limited Trial (*n* = 69)	Notes Without Documentation of a Time-Limited Trial (*n* = 5406)
Who conducted goals of care conversation			
Primary team only	2131 (39)	35 (51)	2096 (39)
Primary team and palliative	976 (18)	13 (19)	963 (18)
Palliative only	2368 (43)	21 (30)	2347 (43)
Patient/family/friends present			
Patient present with or without family/friends	3220 (59)	29 (42)	3191 (59)
Only patient present	1136 (21)	5 (7)	1131 (21)
Only family/friends present	2215 (40)	35 (51)	2180 (40)
Surrogate documented	3006 (55)	30 (43)	2976 (55)
Patient values documented	4951 (90)	67 (97)	4884 (90)
Prognosis documented	4322 (79)	65 (94)	4257 (79)
Change in code status	1971 (36)	15 (22)	1957 (36)
Quantity (percent).			

Quantity (percent).

**Table 3:** Goals of Care Note Documentation of Essential Elements to Consider and Plan a Time-Limited Trial

Time-Limited Trial Component^a^		Notes With Documentation of a Time-Limited Trial (*n* = 69)
1	Discuss patient’s goals and priorities	67 (97)
2	Discuss patient’s prognosis	4257 (79)
	Best case scenario documented	12 (17)
	Worst case scenario documented	8 (12)
	Length of prognosis hours to days	11 (16)
	Length of prognosis days to weeks	9 (3)
	Length of prognosis weeks to months	6 (9)
	Length of prognosis uncertain or no answer	47 (68)
	Predicted functional status expected to return to baseline	4 (6)
	Predicted functional status high likelihood of dependency	29 (42)
	Patient/surrogate agrees with prognosis provided	44 (64)
	Patient/surrogate disagrees with prognosis provided	1 (1)
3	Meet with patient and/or surrogate to discuss a time-limited trial	69 (100)
4A	Define planned duration	66 (96)
	Average trial duration in days (median, range)	2 (0.15–365)
4B	Define clinical criteria of improvement and/or deterioration	68 (99)
	Improved mentation	16 (23)
	Improved respiratory status	12 (17)
	Decreased life-support requirements (e.g., vasopressors)	9 (13)
	Improved mobility	6 (9)
	Evaluated test results	3 (4)
	Improved pain control	2 (3)
5	Discuss which therapies are acceptable to the patient during the trial	62 (90)
	Trial of diet	4 (6)
	Trial of dialysis	3 (4)
	Trial of mechanical ventilation	2 (3)
	Trial of skilled nursing facility	1 (1)
6	Discuss potential decisions to be made at the end of the trial	50 (72)
7	Provide opportunity for patient and/or surrogate to agree or decline time-limited trial	32 (46)
8	Schedule future meeting with patient and/or surrogate to discuss the patient’s response to therapy	35 (51)
9	Document the meeting and plans in the health record	69 (100)

Quantity (percent).

a Adapted from defining the time-limited trial of patients with critical illness: An Official American Thoracic Society Workshop Report, 2024.

## Key Message

This multicenter study found that goals of care notes rarely document time-limited trial use, despite an explicit prompt to document these trials within the note. This finding may represent a substantial gap between time-limited trials being conducted in clinical practice and what is being documented in the electronic health record.
