# Supplementary material for: Characterizing the Documentation of Time-Limited Trials in Goals of Care Notes
Source: J Pain Symptom Manage. Author manuscript; Available in PMC 2025 Aug 19. (PMC12363427; doi:10.1016/j.jpainsymman.2025.04.007)
Supplement: 1 [file NIHMS2101931-supplement-1.pdf]

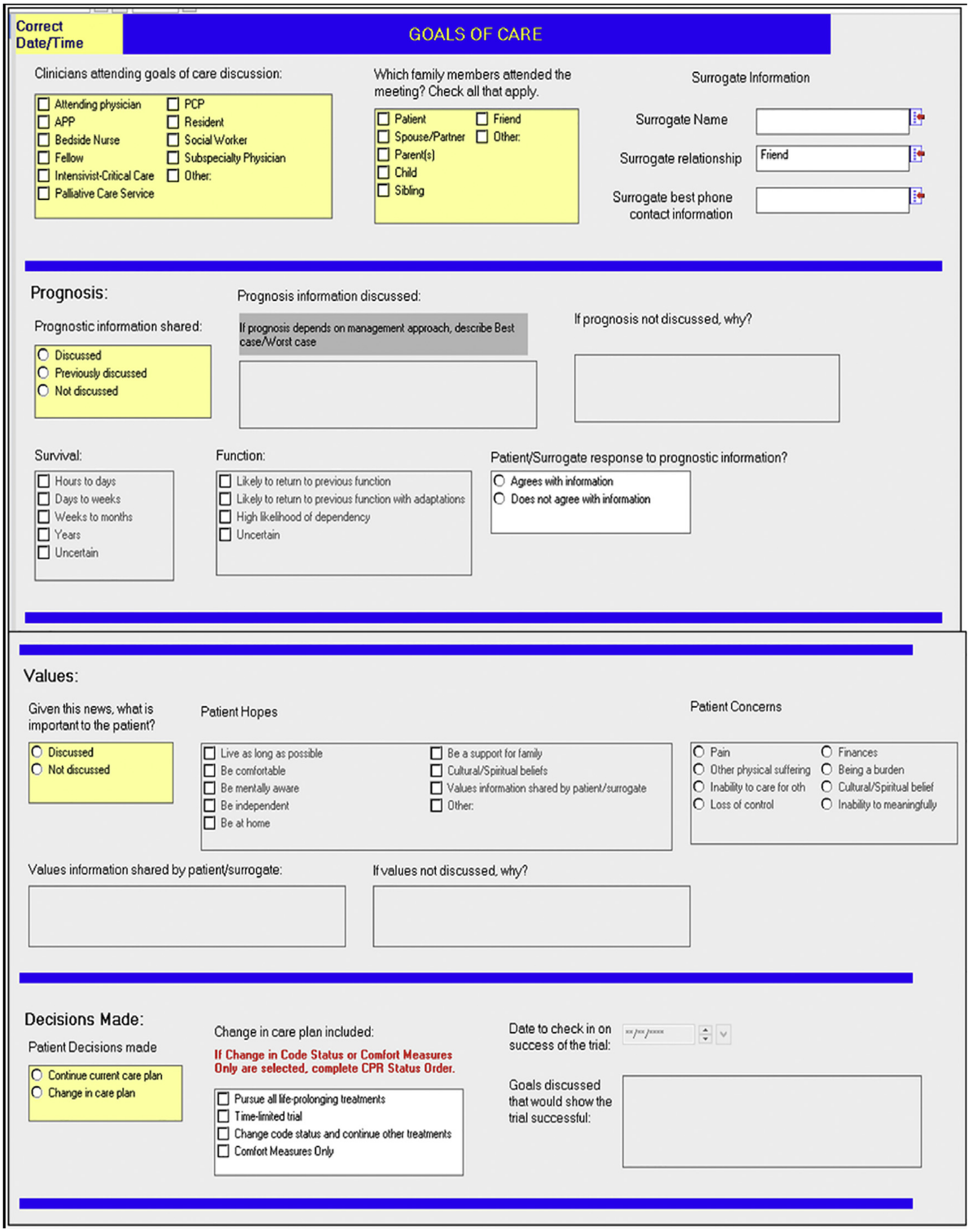

Correct Date/Time

GOALS OF CARE

Clinicians attending goals of care discussion:

- ☐ Attending physician
- ☐ APP
- ☐ Bedside Nurse
- ☐ Fellow
- ☐ Intensivist-Critical Care
- ☐ Palliative Care Service
- ☐ PCP
- ☐ Resident
- ☐ Social Worker
- ☐ Subspecialty Physician
- ☐ Other:

Which family members attended the meeting? Check all that apply.

- ☐ Patient
- ☐ Spouse/Partner
- ☐ Parent(s)
- ☐ Child
- ☐ Sibling
- ☐ Friend
- ☐ Other:

Surrogate Information

Surrogate Name

Surrogate relationship: Friend

Surrogate best phone contact information

Prognosis:

Prognostic information shared:

- ○ Discussed
- ○ Previously discussed
- ○ Not discussed

Prognosis information discussed:

If prognosis depends on management approach, describe Best case/Worst case

If prognosis not discussed, why?

Survival:

- ☐ Hours to days
- ☐ Days to weeks
- ☐ Weeks to months
- ☐ Years
- ☐ Uncertain

Function:

- ☐ Likely to return to previous function
- ☐ Likely to return to previous function with adaptations
- ☐ High likelihood of dependency
- ☐ Uncertain

Patient/Surrogate response to prognostic information?

- ○ Agrees with information
- ○ Does not agree with information

Values:

Given this news, what is important to the patient?

- ○ Discussed
- ○ Not discussed

Patient Hopes

- ☐ Live as long as possible
- ☐ Be comfortable
- ☐ Be mentally aware
- ☐ Be independent
- ☐ Be at home
- ☐ Be a support for family
- ☐ Cultural/Spiritual beliefs
- ☐ Values information shared by patient/surrogate
- ☐ Other:

Patient Concerns

- ○ Pain
- ○ Other physical suffering
- ○ Inability to care for oth
- ○ Loss of control
- ○ Finances
- ○ Being a burden
- ○ Cultural/Spiritual belief
- ○ Inability to meaningfully

Values information shared by patient/surrogate:

If values not discussed, why?

Decisions Made:

Patient Decisions made

- ○ Continue current care plan
- ○ Change in care plan

Change in care plan included:

If Change in Code Status or Comfort Measures Only are selected, complete CPR Status Order.

- ☐ Pursue all life-prolonging treatments
- ☐ Time-limited trial
- ☐ Change code status and continue other treatments
- ☐ Comfort Measures Only

Date to check in on success of the trial: **/**/****

Goals discussed that would show the trial successful:

eFig. 1. Goals of care note template.
